# Supplementary material for: Individual stochasticity in the life history strategies of animals and plants
Source: PLoS One. 2022 Sep 23;17(9):e0273407. doi: 10.1371/journal.pone.0273407 (PMC9506618; doi:10.1371/journal.pone.0273407)
Supplement: S1 Appendix — Contains further information regarding calculations of demographic outcomes, distributions of summary statistics and correlations among life history outcomes in our sample. (PDF) [file pone.0273407.s001.pdf]

1 Supporting Information 1: Appendix

The calculations and analyses in this study were performed in R v3.5.3. The R package "*pracma*" was used to estimate the conditional number of matrices and, where necessary, pseudo-invert these in the calculations (1).

1.1 Measures of variance and uncertainty

The results of the calculations of life history outcomes are typically a set of vectors whose entries give the moments of some demographic outcome ($\boldsymbol{\xi}$) for individuals starting in each stage. Let $\boldsymbol{\xi}_m$ denote the vector for the mth moments. Then the variances are given by the vector

$$V(\boldsymbol{\xi}) = \boldsymbol{\xi}_2 - \boldsymbol{\xi}_1 \circ \boldsymbol{\xi}_1 \tag{1}$$

The skewness and kurtosis are most easily written in terms of the vectors of moments around the mean

$$\hat{\boldsymbol{\xi}}_2 = \boldsymbol{\xi}_2 - \boldsymbol{\xi}_1 \circ \boldsymbol{\xi}_1 \tag{2}$$

$$\hat{\boldsymbol{\xi}}_3 = \boldsymbol{\xi}_3 - 3(\boldsymbol{\xi}_1 \circ \boldsymbol{\xi}_2) + 2(\boldsymbol{\xi}_1 \circ \boldsymbol{\xi}_1 \circ \boldsymbol{\xi}_1) \tag{3}$$

$$\hat{\boldsymbol{\xi}}_4 = \boldsymbol{\xi}_4 - 4(\boldsymbol{\xi}_1 \circ \boldsymbol{\xi}_3) + 6(\boldsymbol{\xi}_1 \circ \boldsymbol{\xi}_1 \circ \boldsymbol{\xi}_2) - 3(\boldsymbol{\xi}_1 \circ \boldsymbol{\xi}_1 \circ \boldsymbol{\xi}_1 \circ \boldsymbol{\xi}_1) \tag{4}$$

In terms of these central moments, the vectors giving the skewness and excess kurtosis of the elements of $\boldsymbol{\xi}$ are given by

$$Sk(\boldsymbol{\xi}) = \mathcal{D}\left(\hat{\xi}_2\right)^{-3/2} \hat{\boldsymbol{\xi}}_3 \tag{5}$$

$$\kappa(\boldsymbol{\xi}) = \mathcal{D}\left(\hat{\boldsymbol{\xi}}_2 \circ \hat{\boldsymbol{\xi}}_2\right)^{-1} \hat{\boldsymbol{\xi}}_4 - 3 \tag{6}$$

The skewness quantifies the asymmetry of the distribution; positive values indicate an extended positive tail, negative values indicate the opposite. Skewness can be interpreted as a measure of inequality. Kurtosis is a measure of the extent of extreme values, measured relative to the normal distribution. Positive kurtosis indicates a distribution with heavier tails (leptokurtic) and thus more likely to exhibit extreme values, either positive or negative.

1.2 Calculation of individual stochasticity.

The calculations of individual stochasticity rely on the Markov chain transition matrix $\mathbf{U}$ and the fundamental matrix

$$\mathbf{N} = (\mathbf{I} - \mathbf{U})^{-1}. \tag{7}$$

The (i, j) entry of $\mathbf{N}$ is the mean time spent in stage i, prior to absorption (i.e., death), of an individual starting in stage j.

1.2.1 Longevity

Longevity of an individual is calculated as the sum of the time spent in every transient stage, until eventual absorption (2). The first four moments of longevity are given in (3),

$$
\begin{aligned}
\boldsymbol{\eta}_1^\mathsf{T} &= \mathbf{1}_\tau \mathbf{N} && (8)\\
\boldsymbol{\eta}_2^\mathsf{T} &= \boldsymbol{\eta}_1^\mathsf{T} (2\mathbf{N} - \mathbf{I}) && (9)\\
\boldsymbol{\eta}_3^\mathsf{T} &= \boldsymbol{\eta}_1^\mathsf{T} \left(6\mathbf{N}^2 - 6\mathbf{N} + \mathbf{I}\right) && (10)\\
\boldsymbol{\eta}_4^\mathsf{T} &= \boldsymbol{\eta}_1^\mathsf{T} \left(24\mathbf{N}^3 - 36\mathbf{N}^2 + 14\mathbf{N} - \mathbf{I}\right) && (11)
\end{aligned}
$$

The ith entry of $\boldsymbol{\eta}_m$ is the mth moment of longevity for an individual starting in stage i.

1.2.2 Lifetime reproductive output (LRO)

Calculation of LRO requires reward matrices whose entries give the moments of the reproductive output reward associated with each transition. Let $\mathbf{f}$ be a vector giving the mean reproductive output of each stage. If a single type of offspring is produced $\mathbf{f}^\mathsf{T}$ is the first row of the fertility matrix $\mathbf{F}$. For species that produce multiple types of offspring (i.e., in which $\mathbf{F}$ contains positive entries in more than one row), we summed all types at each age and treated the sum as the mean stage-specific reproductive output. Modeling the number of offspring as a Poisson random variable with that mean gives the reward matrices

$$
\begin{aligned}
\mathbf{R}_1 &= \mathbf{1}_s \mathbf{f}^\mathsf{T} \mathbf{Z} && (12)\\
\mathbf{R}_2 &= \mathbf{R}_1 + (\mathbf{R}_1 \circ \mathbf{R}_1) && (13)\\
\mathbf{R}_3 &= \mathbf{R}_1 + 3(\mathbf{R}_1 \circ \mathbf{R}_1) + (\mathbf{R}_1 \circ \mathbf{R}_1 \circ \mathbf{R}_1) && (14)\\
\mathbf{R}_4 &= \mathbf{R}_1 + 7(\mathbf{R}_1 \circ \mathbf{R}_1) + 6(\mathbf{R}_1 \circ \mathbf{R}_1 \circ \mathbf{R}_1) \\
&\quad + (\mathbf{R}_1 \circ \mathbf{R}_1 \circ \mathbf{R}_1 \circ \mathbf{R}_1). && (15)
\end{aligned}
$$

Following Theorem 1 of (4), we write $\tilde{\boldsymbol{\rho}}_k$ for the vector, of dimension $\tau \times 1$ of the kth moments of lifetime reproduction for individuals starting in each transient stage. We define the matrix

$$
\mathbf{Z} = \left(\begin{array}{c|c} \mathbf{I}_{\tau\times\tau} & \mathbf{0}_{\tau\times\alpha} \end{array} \right); \tag{16}
$$

and also define $\tilde{\mathbf{R}}_k$, the $\tau \times \tau$ submatrix of $\mathbf{R}_k$ corresponding to transitions among the transient states:

$$
\tilde{\mathbf{R}}_k = \mathbf{Z}\mathbf{R}_k\mathbf{Z}^\mathsf{T}. \tag{17}
$$

In terms of these quantities, the first four moments of LRO are

$$
\begin{aligned}
\tilde{\boldsymbol{\rho}}_1 &= \mathbf{N}^\mathsf{T}\mathbf{Z}(\mathbf{P} \circ \mathbf{R}_1)^\mathsf{T} \mathbf{1}_s && (18)\\
\tilde{\boldsymbol{\rho}}_2 &= \mathbf{N}^\mathsf{T}\left[\mathbf{Z}(\mathbf{P} \circ \mathbf{R}_2)^\mathsf{T} \mathbf{1}_s + 2\left(\mathbf{U} \circ \tilde{\mathbf{R}}_1\right)^\mathsf{T} \tilde{\boldsymbol{\rho}}_1\right] && (19)\\
\tilde{\boldsymbol{\rho}}_3 &= \mathbf{N}^\mathsf{T}\left[\mathbf{Z}(\mathbf{P} \circ \mathbf{R}_3)^\mathsf{T} \mathbf{1}_s + 3\left(\mathbf{U} \circ \tilde{\mathbf{R}}_2\right)^\mathsf{T} \tilde{\boldsymbol{\rho}}_1 + 3\left(\mathbf{U} \circ \tilde{\mathbf{R}}_1\right)^\mathsf{T} \tilde{\boldsymbol{\rho}}_2\right] && (20)\\
\tilde{\boldsymbol{\rho}}_4 &= \mathbf{N}^\mathsf{T}\left[\mathbf{Z}(\mathbf{P} \circ \mathbf{R}_4)^\mathsf{T} \mathbf{1}_s + 4\left(\mathbf{U} \circ \tilde{\mathbf{R}}_3\right)^\mathsf{T} \tilde{\boldsymbol{\rho}}_1\right. \\
&\quad \left. +6\left(\mathbf{U} \circ \tilde{\mathbf{R}}_2\right)^\mathsf{T} \tilde{\boldsymbol{\rho}}_2 + 4\left(\mathbf{U} \circ \tilde{\mathbf{R}}_1\right)^\mathsf{T} \tilde{\boldsymbol{\rho}}_3\right]. && (21)
\end{aligned}
$$

1.2.3 Age at maturity.

The age at maturity is defined as the time to first enter any stage defined as reproductive, that is any stage j for which column j of $\mathbf{F}$ is non-zero. The technique, detailed in Section 5.3.3 in (5) has two steps. First, the transition matrix is modified to make the reproductive stages absorbing. An individual will end in one or the other of the two absorbing states, death-before-reproduction or reproduction-before-death. Then a conditional Markov chain is constructed, conditional on reaching reproduction. The age at maturity is the time to absorption in this conditional chain. We calculated the mean, standard deviation, and coefficient of variation of this time.

1.2.4 Generation time.

The offspring production at age x of an individual starting in stage j is given by the vector

$$\mathbf{m}^{(j)}(x) = \mathcal{D}\left(\mathbf{F}\mathbf{N}\mathbf{e}_j\right)^{-1}\left(\mathbf{F}\mathbf{U}^x\mathbf{e}_j\right) \tag{22}$$

where the entries of $\mathbf{m}^{(j)}$ correspond to different types of offspring (2) .

The cohort generation time, given by the mean of this distribution, is

$$\begin{aligned}
\boldsymbol{\mu}_1^{(j)} &= \sum_x x\mathbf{m}^{(j)}(x) & (23)\\
&= \mathcal{D}\left(\mathbf{F}\mathbf{N}\mathbf{e}_j\right)^{-1}\mathbf{F}\left(\sum_x x\mathbf{U}^x\right)\mathbf{e}_j & (24)\\
&= \mathcal{D}\left(\mathbf{F}\mathbf{N}\mathbf{e}_j\right)^{-1}\mathbf{F}\mathbf{N}\mathbf{U}\mathbf{N}\mathbf{e}_j, & (25)
\end{aligned}$$

as in (2).

1.2.5 Extent of iteroparity

Considering previous work estimating the extent of iteroparity, which focuses on the age dispersion of reproduction (6), we used the distribution $\mathbf{m}^{(j)}$ in (22) to derive a new result for the variance of the ages of mothers at the birth of offspring. The second moment of that age is

$$\boldsymbol{\mu}_2^{(j)} = \mathcal{D}\left(\mathbf{F}\mathbf{N}\mathbf{e}_j\right)^{-1}\mathbf{F}\left(\sum_x x^2\mathbf{U}^x\right)\mathbf{e}_j \tag{26}$$

The new part of this expression is $\sum_x x^2\mathbf{U}^x$. We can write and then simplify this summation,

$$\begin{aligned}
\sum_x x^2\mathbf{U}^x &= \mathbf{0} + \mathbf{U} + 4\mathbf{U}^2 + 9\mathbf{U}^3 + \cdots & (27)\\
&= \mathbf{0} + \mathbf{U} + 2\mathbf{U}^2 + 3\mathbf{U}^3 + \cdots \\
&\quad +2\mathbf{U}^2 + 6\mathbf{U}^3 + 12\mathbf{U}^4 + \cdots & (28)\\
&= \mathbf{N}\mathbf{U}\mathbf{N} + \mathbf{U}\left(2\mathbf{U} + 6\mathbf{U}^2 + 12\mathbf{U}^3 + \cdots\right) & (29)\\
&= \mathbf{N}\mathbf{U}\mathbf{N} + \mathbf{U}\sum_x x(x+1)\mathbf{U}^x & (30)\\
&= (\mathbf{I}+\mathbf{U})\mathbf{N}\mathbf{U}\mathbf{N} + \mathbf{U}\sum_x x^2\mathbf{U}^x. & (31)
\end{aligned}$$

Solving for $\sum_x x^2\mathbf{U}^x$, which appears on both sides, yields

$$\sum_x x^2\mathbf{U}^x = \mathbf{N}(\mathbf{I}+\mathbf{U})\mathbf{NUN} \tag{32}$$

and thus

$$\boldsymbol{\mu}_2^{(j)} = \mathcal{D}\left(\mathbf{FNe}_j\right)^{-1}\mathbf{FN}(\mathbf{I}+\mathbf{U})\mathbf{NUNe}_j \tag{33}$$

The vector of variances is given by

$$V(\boldsymbol{\mu}^{(j)}) = \boldsymbol{\mu}_2^{(j)} - \boldsymbol{\mu}_1^{(j)} \circ \boldsymbol{\mu}_1^{(j)} \tag{34}$$

and from this we calculated the standard deviation and finally the coefficient of variation of the age of production of offspring. The coefficient of variation is dimensionless and is thus appropriate for comparing across life histories of different absolute lengths.

1.3 Interspecific variation in statistics of longevity

Similar to the distributions for LRO in Figure 1, we show the distribution of the statistics of longevity for all plant and animal populations in Figure 2. We show trimmed histograms for some of the statistics for plants. Mean longevity, for example, has a high number of populations with a life expectancy of about 1. Paired with a long tail of extreme lifespans (up to a life expectancy of 150), the body of the distribution would be completely obscured if we showed the full range. The same is true for standard deviation, and kurtosis in longevity.

Plants show more variability among populations than animals do. They have a larger range in the values observed for each of the longevity statistics. All of these statistics indicate that plants experience greater variation and uncertainty in their life lifespans than animals do, although extreme examples can be found in both subsets.

1.4 Correlations between life history outcomes

In figures 3 and 4, we show the Pearson product-moments correlations of all 16 of the demographic outcomes we include in our analyses for animals and plants, respectively. Some of these statistics are very tightly correlated; others not at all.

2 Bibliography

[1] Borchers, Hans W. pracma: Practical Numerical Math Functions R package version 2.3.8.; 2022 Available from: https://CRAN.R-project.org/package=pracma.

[2] Caswell H. Stage, age and individual stochasticity in demography. Oikos. 2009;118:1763–1782.

[3] Caswell H. Sensitivity Analysis: Matrix Methods in Demography and Ecology. Springer Nature; 2019.

[4] van Daalen SF, Caswell H. Lifetime reproductive output: individual stochasticity, variance, and sensitivity analysis. Theoretical Ecology. 2017;10(3):355–374.

[5] Caswell H. Matrix population models: Construction, analysis, and interpretation. 2nd ed. Sunderland: Sinauer Associates; 2001.

[6] Steiner, Ulrich K and Tuljapurkar, Shripad and Coulson, Tim. Generation time, net reproductive rate, and growth in stage-age-structured populations The American Naturalist. 2014;183(6):771–783.

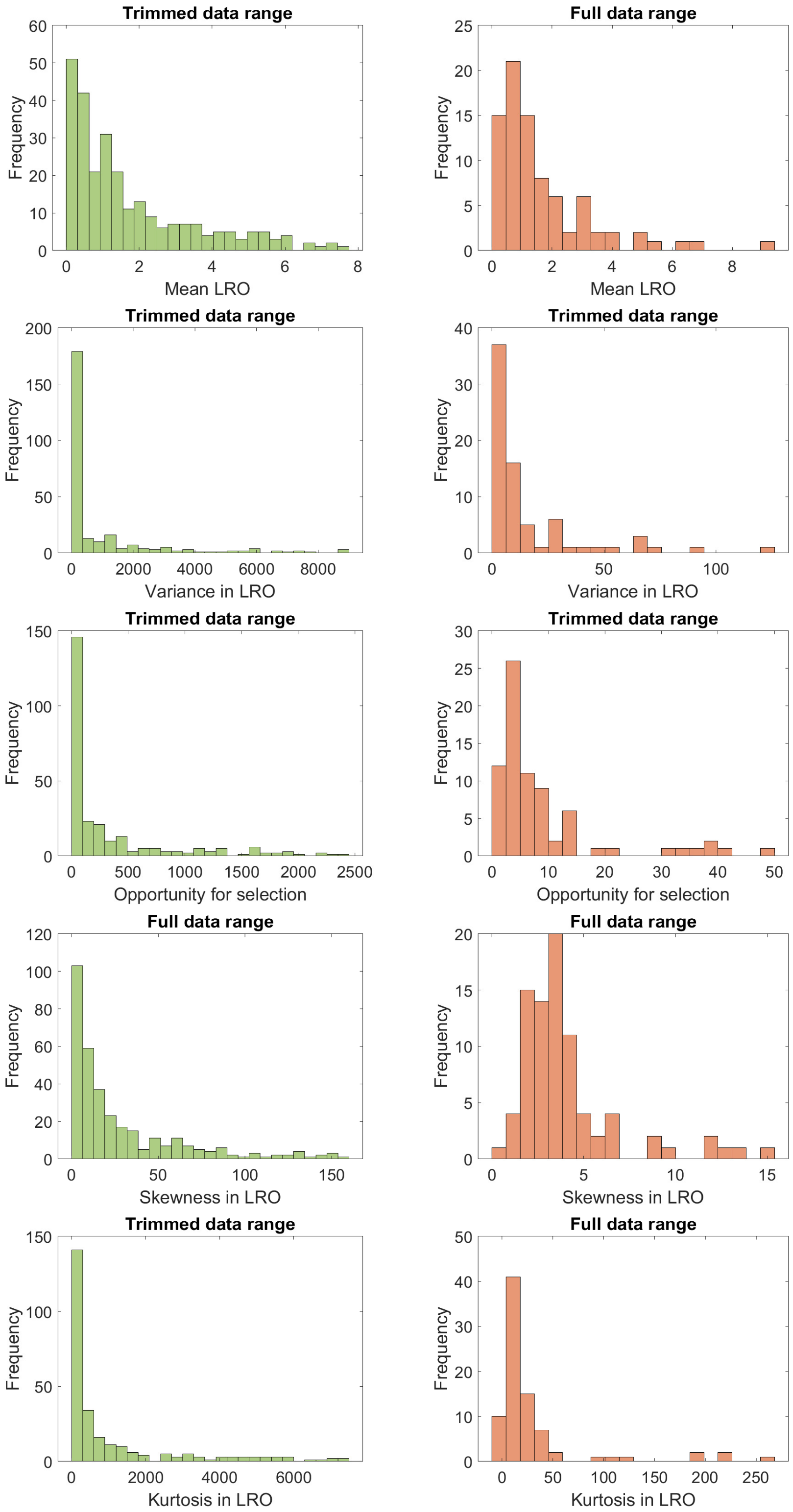

Figure 1: Histograms of the statistics of LRO (mean, variance, OFS, skewness, and kurtosis) for 332 populations of plants (left hand figures), and for 83 populations of animals (right hand figures). For both, some distributions were trimmed to better show the shape of the distribution; where the top of the plot reads "Trimmed data range" we left out 20% of the values for animal and plant populations (highest and lowest 10%).

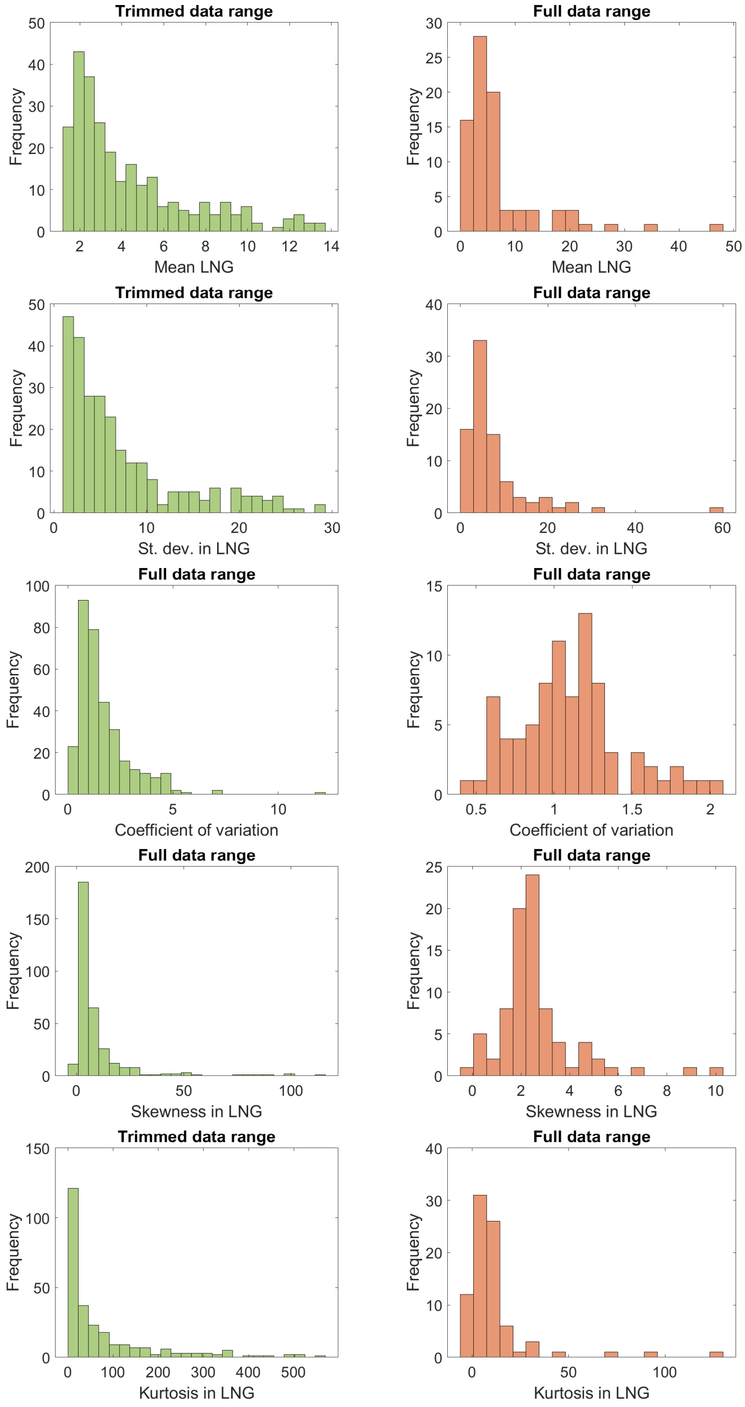

Figure 2: Histograms of the statistics of longevity (mean, standard deviation, coefficient of variation, skewness, and kurtosis) for 332 species of plants (left hand figures), and for 83 species of animals (right hand figures). For both, some distributions were trimmed to better show the shape of the distribution; where the top of the plot reads "Trimmed data range" we left out 20% of the values for animal and plant populations (highest and lowest 10%).

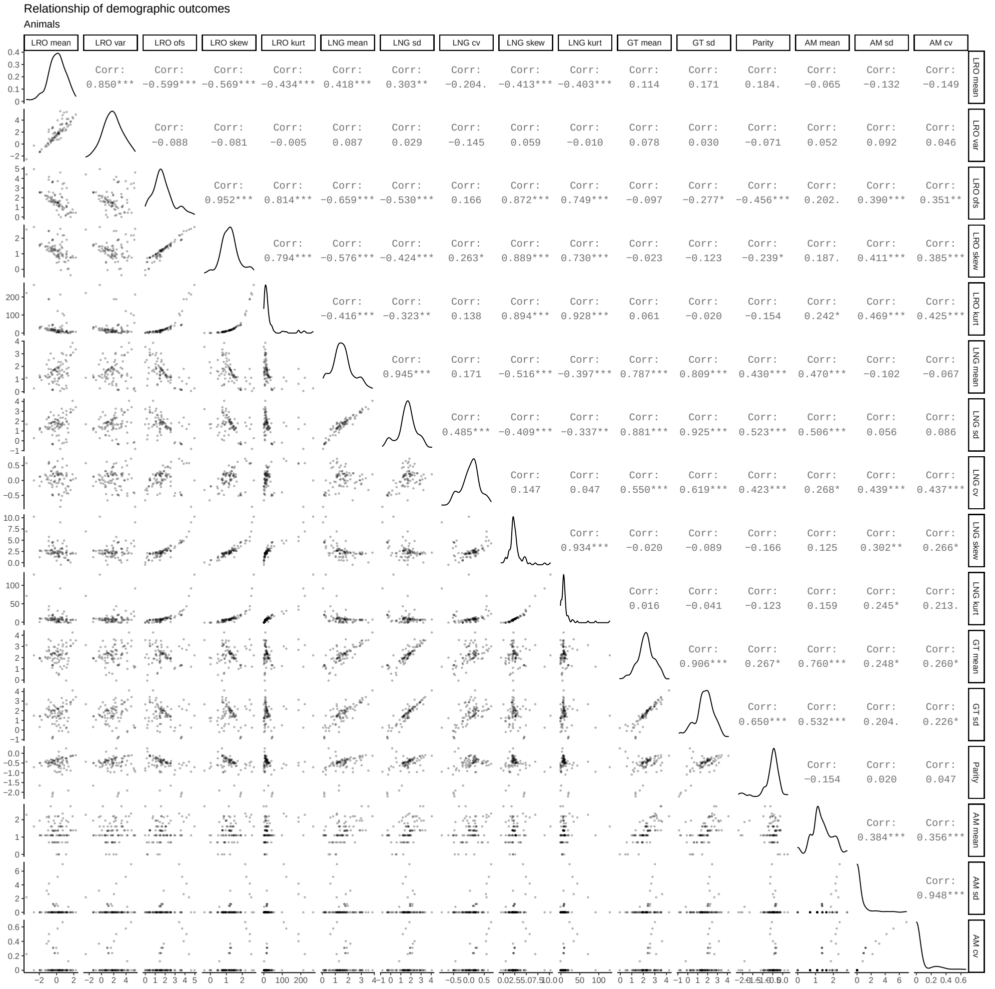

Figure 3: Relationship among the demographic outcomes in animals. Lifetime reproductive output (LRO), longevity (LNG), generation time (GT), modes of parity (Parity), and age at maturity (AM) were log-transformed, with the exception of the kurtosis of LRO, the kurtosis and skewness of longevity, and measures of variability in age at maturity. Pearson correlation coefficients are in the upper diagonal of the matrix while scatterplots are located in the lower diagonal. Finally, density plots are shown on the diagonal to visualize the distribution of the demographic outcomes.

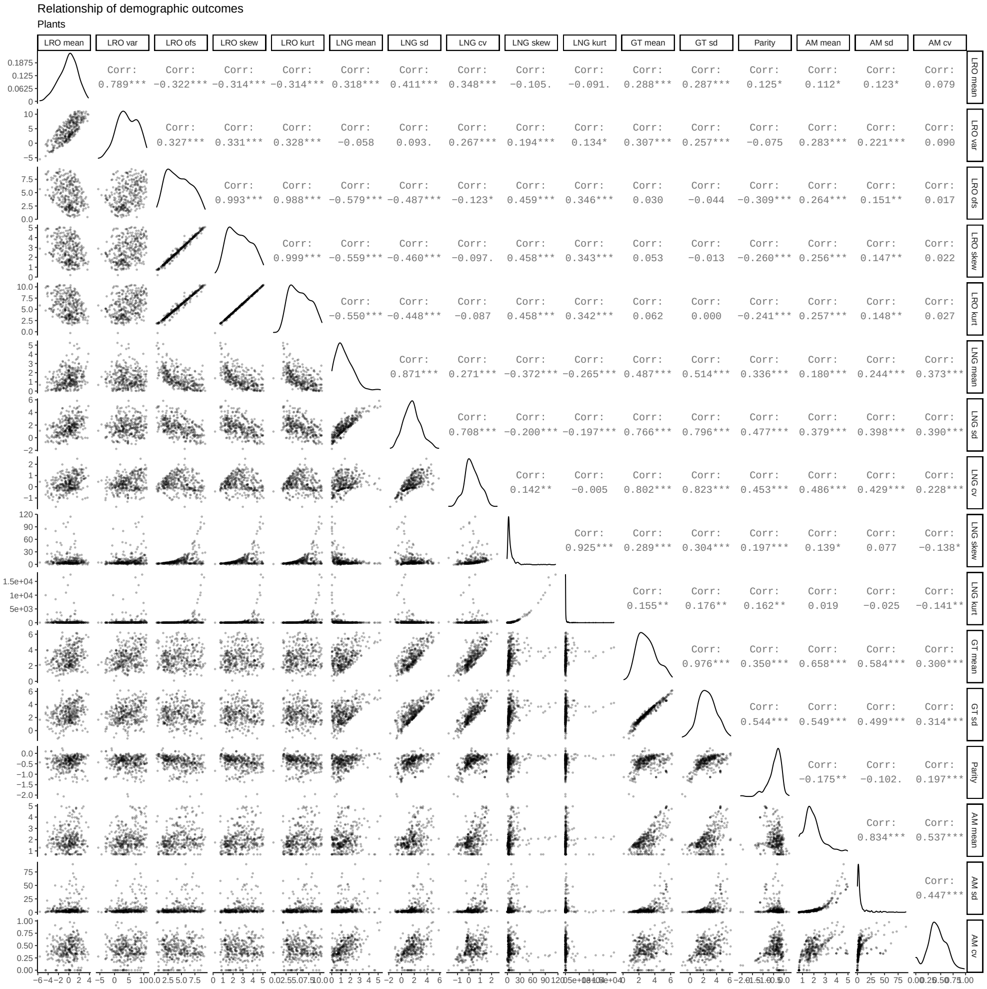

Figure 4: Relationship among the demographic outcomes in plants. Lifetime reproductive output (LRO), longevity (LNG), generation time (GT), modes of parity (Parity), and age at maturity (AFR) were log-transformed, with the exception of the kurtosis and skewness of longevity and measures of variability in age at maturity. Pearson correlation coefficients are in the upper diagonal of the matrix while scatterplots are located in the lower diagonal. Finally, density plots are shown on the diagonal to visualize the distribution of the demographic outcomes.
